# Cumulative Effects of Physical Activity, Dietary Variety, and Social Participation on Active Life Expectancy

**DOI:** 10.1093/geroni/igaa057.603

**Published:** 2020-12-16

**Authors:** Satoshi Seino, Akihiko Kitamura, Yui Tomine, Mariko Nishi, Yu Nofuji, Yuri Yokoyama, Hidenori Amano, Shoji Shinkai

**Affiliations:** 1 Tokyo Metropolitan Institute of Gerontology, Itabashi-ku, Japan; 2 Tokyo Metropolitan Institute of Gerontology, Itabashi, Japan; 3 Tokyo Metropolitan Institute of Gerontology, Itabashi-ku, Tokyo, Japan; 4 Tokyo Metropolitan Institute of Gerontology, Tokyo, Japan

## Abstract

Regular physical activity, dietary variety, and active social participation are modifiable and influential factors of adverse health outcomes. However, the cumulative effects of these behaviors are unknown. We examined these cumulative associations with active life loss in older adults. We analyzed 3-year longitudinal data from 7246 initially non-disabled residents aged 65-84 years from 18 districts of Ota City, Tokyo. Sufficiency of moderate- to vigorous-intensity physical activity (MVPA) of ≥150 minutes/week, dietary variety score (DVS) of ≥3, and social participation of ≥1 time/month were assessed using self-administered questionnaires. We operationally defined active life loss for individuals as being newly certified for long-term care insurance or death without prior certification. Multilevel survival analyses were applied to calculate hazard ratios (HRs) and 95% confidence intervals (CIs). During an average follow-up of 2.9 years, the cumulative incidence of active life loss was 11.3% (817 individuals: 650 new certifications and 167 deaths without prior certification). Multivariate-adjusted HRs (95% CIs) for active life loss were 0.73 (0.58-0.92) in only MVPA of ≥150 minutes/week, 0.88 (0.67-1.15) in only DVS of ≥3, 0.75 (0.51-1.09) in only social participation of ≥1 time/month, 0.56 (0.45-0.70) in the group satisfying any two, and 0.52 (0.40-0.67) in the group satisfying all three behaviors, compared with a reference group that did not satisfy any of the behaviors. Sensitivity analysis that excluded active life losses during the first year showed similar results. The combination of regular physical activity, dietary variety, and social participation further enhances the effects on active life expectancy than individual practices.

